# Cobalt‐Doped ZnO Nanocomposits for Efficient Dye Degradation: Charge Transfer

**DOI:** 10.1002/open.202400203

**Published:** 2024-09-09

**Authors:** Buzuayehu Abebe, Bontu Kefale, Guta Amenu, Leta Guta, C. R. Ravikumar, Taymour A. Hamdalla, S. Giridhar Reddy, Dereje Tsegaye, H. C. Ananda Murthy

**Affiliations:** ^1^ Department of Applied Chemistry Adama Science and Technology University P.O. Box 1888 Adama Ethiopia; ^2^ Department of Science East-West Institute of Technology Bangalore 560091 India; ^3^ Department of Physics University of Tabuk KSA; ^4^ Department of Chemistry Amrita School of Engineering Amrita Vishwa Vidyapeetham Bengaluru 560035 India; ^5^ School of Applied Sciences Papua New Guinea University of Technology Lae Morobe Province 411 Papua New Guinea

**Keywords:** nanomaterial, Doping, Photocatalysis, methylene blue, charge transfer

## Abstract

Doping enhances the optical properties of high‐band gap zinc oxide nanoparticles (ZnO NPs), essential for their photocatalytic activity. We used the combustion approach to synthesize cobalt‐doped ZnO heterostructure (CDZO). By creating a mid‐edge level, it was possible to tune the indirect band gap of the ZnO NPs from 3.1 eV to 1.8 eV. The red shift and reduction in the intensity of the photoluminescence (PL) spectra resulted from hindrances in electron‐hole recombination and sp‐d exchange interactions. These improved optical properties expanded the absorption of solar light and enhanced charge transfer. The field emission scanning electron microscopy (FESEM) image and elemental mapping analysis confirmed the CDZO′s porous nature and the dopant‘s uniform distribution. The porosity, nanoscale size (25–55 nm), and crystallinity of the CDZO were further verified by high‐resolution transmission electron microscopy (HRTEM) and selected area electron image analysis. The photocatalytic activity of the CDZO exhibited much greater efficiency (*k*=0.131 min^−1^) than that of ZnO NPs (*k*=0.017 min^−1^). Therefore, doped heterostructures show great promise for industrial‐scale environmental remediation applications.

## Graphical abstract

The combustion approach produces porous, crystalline cobalt‐doped ZnO material with good cobalt surface distribution. The material also has great potential for the degradation of methylene blue dye.

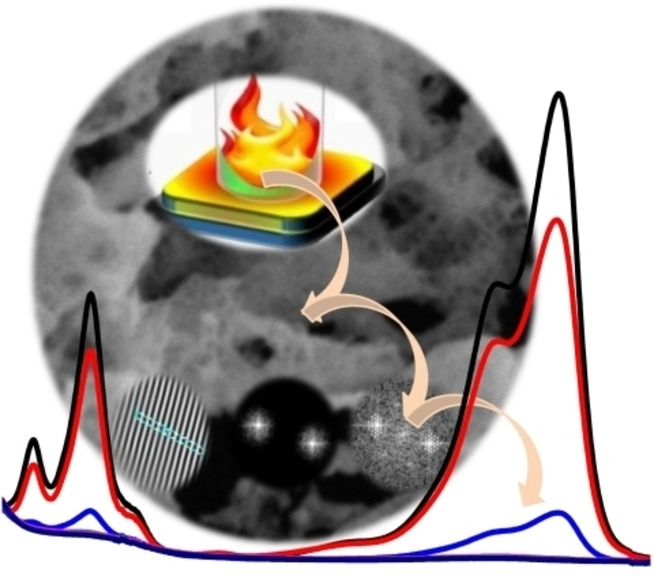

## Introduction

The advanced oxidation process removes organic pollutants by reacting with hydroxyl radicals. This process has potential applications in catalysis and medicine.[^1^, ^2^] Nonbiodegradable organic pollutants like methylene blue (MB) can harm human health and aquatic life.[^3^, ^4^] Nanotechnology has led to progress in synthesizing nanomaterials for water treatment, mainly through doping, which can adjust the optical properties of nanoscale metal oxide semiconductors.[^5^, ^6^] Doping of transition metals such as cobalt tunes the optical properties of host materials and harvests visible light.[^7^, ^8^] Cobalt doping changes the optoelectric properties of ZnO NPs due to its redox reaction under photon excitation.^[9]^ The ionic radius of Co (0.58 Å) in the tetrahedral coordination is slightly smaller than that of Zn (0.60 Å). Therefore, including the smaller ionic radius of Co shifts the XRD peaks towards a high angle.^[10]^ The presence of dopant Co ions in the ZnO lattice creates a new Co 3d energy level within the band gap energy of ZnO due to the sp‐d strong exchange interaction.^[11]^ This energy difference allows for various photon‐excited electron‐hole transitions. Electrons can move from the Co 3d level to the conduction band of ZnO, leading to the oxidation of Co from the Co (II) to the Co (III) state. Additionally, electrons can transferred from the Co 3d level to the ZnO valence band, reducing Co(II) to Co (I). The d‐d transition within the Co dopant can also occur within the host band gap,^[12]^ extending the visible light‐harvesting property of ZnO. Furthermore, the development of the ZnO/Co_3_O_4_ n‐p heterostructure has significant advantages for charge transfer through the interface, further increasing the electron‐hole relaxation time without recombination.

Meky *et al*.^[13]^ confirmed the improved visible light absorption property of Co‐doped ZnO heterostructures due to the sp‐d strong exchange interaction. The analysis showed three bands in the visible region due to the Co d‐d transition, observed at wavelengths of 650, 610, and 570 nm associated with specific transitions. The study also reported morphology and defect density changes with increasing Co dopant concentration.

Chen *et al*.^[14]^ also reported these three d‐d transition peaks associated with proper cobalt inclusion in the ZnO lattice without affecting its structure. Similarly, Kazmi *et al*.^[15]^ reported the perfect inclusion of cobalt in the ZnO lattice without affecting its structural integrity or the formation of the Co_3_O_4_ phase. Additionally, the study confirmed the formation of a mid‐state band gap level using ultraviolet photoelectron spectroscopy analysis. Chehhat *et al*.^[16]^ reported a shift in PL spectra wavelength for doped materials compared to single‐host ZnO, linked to the sp‐d strong exchange interaction due to the substitution of zinc for Co. Kaphle *et al*.^[17]^ discussed the use of cobalt mid‐state level as an electron sink, confirmed by the reduction in the emission intensity in the PL spectra. They also reported an increase in the intensity of the broad ZnO visible light emission band with increasing cobalt dopant, indicating the formation of more defects, which was also supported by the study by Chanda *et al.*.^[18]^ These research works reported an improvement in ZnO properties associated with cobalt doping. However, there are gaps in the investigation of methods for producing efficient porous material without complex procedures and with less time, as well as a mechanism that interprets extended light absorption and charge transfer properties. Additionally, LaMer′s nanocrystal growth and HSAB theory should be considered in crystal synthesis procedures.[^19^, ^20^]

This study uses a straightforward bottom‐up combustion method to create pure ZnO and Co_3_O_4_ NPs and Co‐doped ZnO heterostructures. Poly (vinyl alcohol) (PVA) was used as a capping agent during the synthesis of the materials. X‐ray diffraction (XRD) analysis was conducted to confirm the formation of the synthesized materials′ (Co‐doped ZnO/Co_3_O_4_ heterostructures), purity, and crystallinity. FESEM/TEM images and elemental mapping analysis confirmed the porous morphology and distribution of the Co‐dopant. Furthermore, UV‐vis‐DRS and PL analyses compared the heterostructures′ improved optoelectrical properties to single ZnO NPs. The optimized heterostructure also exhibited enhanced photocatalytic potential with a rate constant value of k=0.131 min^−1^, attributed to Co doping.

## Results and Discussion

**Figure 1:**
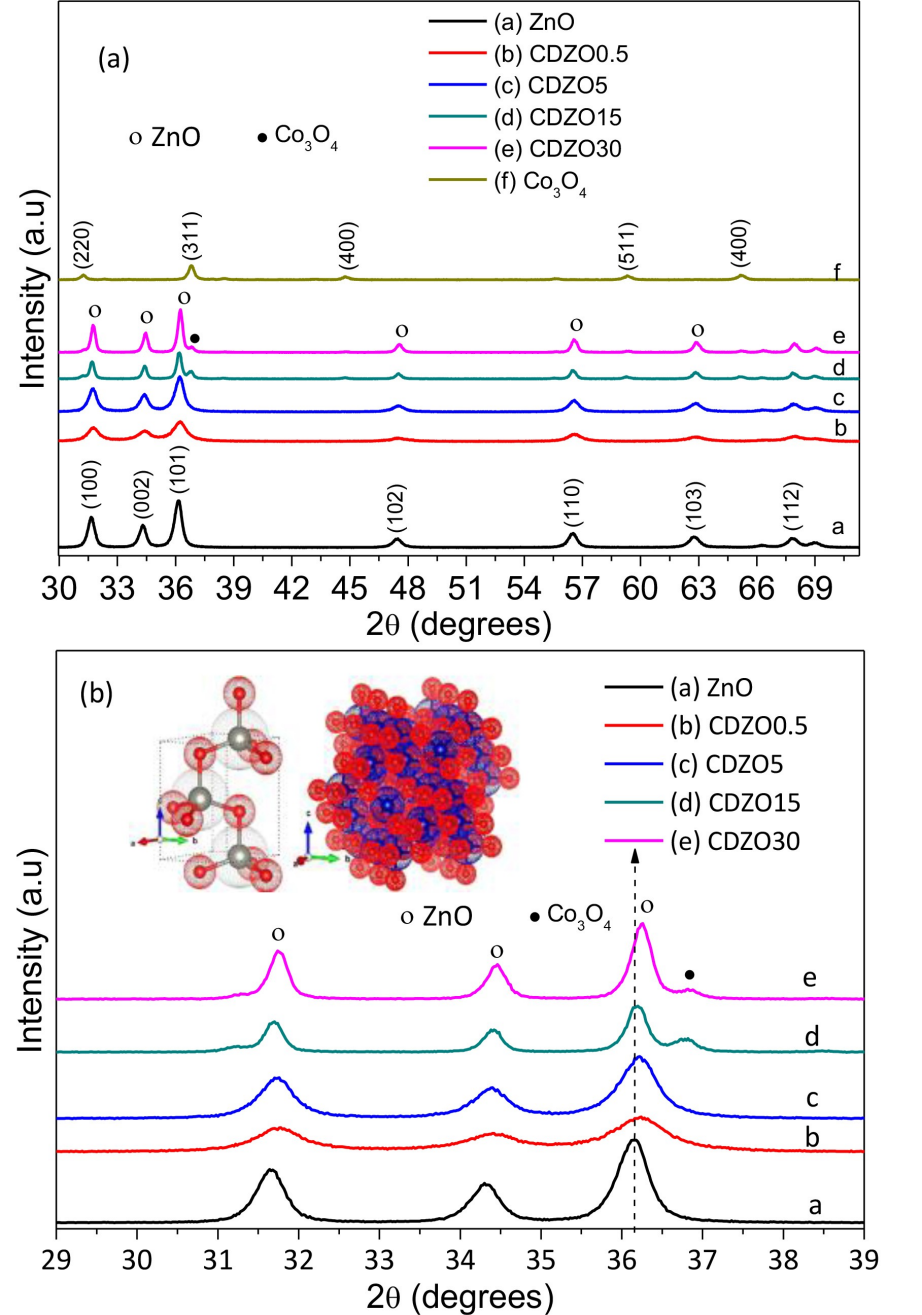
(a) XRD patterns for ZnO and Co_3_O_4_ NPs and Co‐doped ZnO heterostructure. (b) magnified view of ZnO and Co‐doped ZnO heterostructures with different Co percentages. The inset structure is a 3D structure of ZnO and Co_3_O_4_ obtained from VESTA software from AMSED CIF data.

The XRD patterns of ZnO NPs and Co‐doped ZnO heterostructures with different Co percentages are illustrated in Figure 1. The 2θ and respective planes of ZnO crystals show the formation of ZnO crystals (JCPDS# 0036–1451). The diffractogram of Co crystals shows the formation of the fcc Co_3_O_4_ structure (JCPDS# 042–1467). The pattern of the Co‐ZnO crystals also shows the Wurtzite hexagonal structure up to 5 % doping (JCPDS# 0036–1451), indicating that Co perfectly doped the ZnO lattice without forming secondary phases within the detection limit of the XRD instrument. This good solubility without affecting the ZnO structure is related to their near‐comparable ionic size.^[15]^

Of course, the Co ion has a slightly smaller ionic radius than the Zn ion, shifting the XRD peaks to a higher angle.^[10]^ However, by increasing the dopant amount to 15 and 30 %, a Co_3_O_4−_ independent peak appeared. As the cobalt amount increased, the Co_3_O_4_ phase developed on the surface of ZnO as a heterojunction instead of its inclusion in the ZnO lattice.^[21]^ Furthermore, increasing the dopant amount from 15 to 30 % increased the Co_3_O_4_ peak intensity.

**Figure 2:**
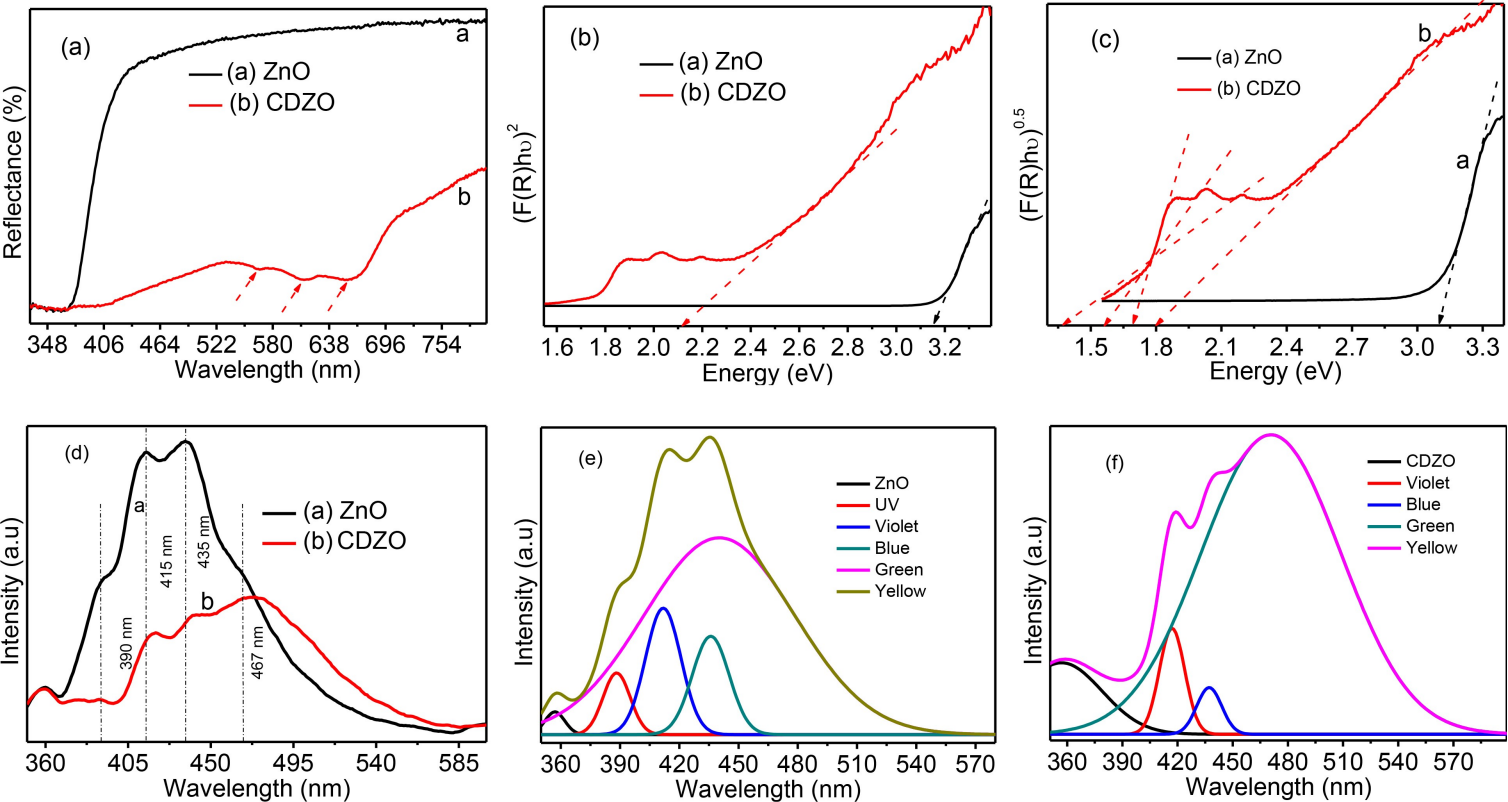
UV‐vis‐DRS and PL optical properties of ZnO NPs and CDZO heterostructure (a) the % reflectance vs. wavelength UV‐vis‐DRS spectra, (b and c) the Kubelka‐Munk plots, (d) PL Spectra, and (e and f) deconvoluted PL spectra of ZnO NPs and CDZO heterostructure.

**Figure 3:**
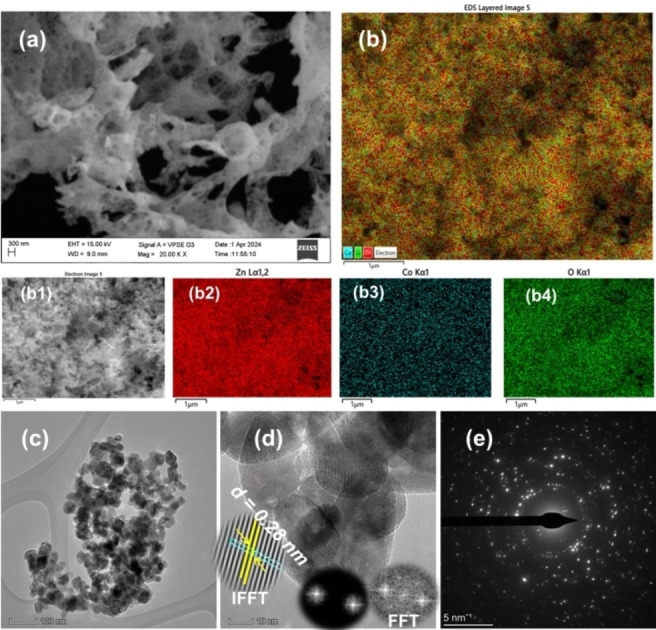
FESEM images of CDZO heterostructure (a) porous morphology, (b) EDS layered image, (b1) electron image, (b2–b4) EDS mapping images of Zn, Co, and O, respectively. (c) TEM image, (d) HRTEM image, (e) SAED ring images. The inset FFT and IFFT in image d represent the fast Fourier and inverse Fourier transform, respectively.

Figure 2(a–c) illustrates the reflectance versus wavelength UV‐vis‐DRS spectra of ZnO and CDZO heterostructures and the direct and indirect Kubelka‐Munk plots, respectively. The CDZO heterostructure exhibits the smallest reflection and three additional absorption bands in the visible region. The reduced reflection in the heterostructures is attributed to their greater visible light absorption property, which is associated with forming mid‐band levels. The three bands in the visible region at wavelengths of 565, 610, and 660 nm correspond to the ^4^A2 to ^2^E (G), ^4^A2 to ^4^T1 (P), and ^4^A2 to ^2^A1 (G) transitions, respectively.^[13]^ The indirect band gap values for these transitions were determined from the Kubelka‐Munk plot and found to be 1.71, 1.55, and 1.35 eV for 565, 610, and 660 nm, respectively. The direct bandgap values for ZnO NPs and CDZO heterostructures are 3.15 and 2.12 eV, respectively (Figure 2c). Furthermore, the indirect band gap values for ZnO and CDZO heterostructures are 3.1 and 1.8 eV, respectively (Figure 3c). The band gap tuning for CDZO is attributed to creating mid‐band levels due to Co inclusion.

The PL intensity for CDZO heterostructure is smaller than that of the ZnO NPs. This emission intensity reduction for CDZO indicates the presence of electron‐hole recombination hindrance properties. The recombination hindrance is the presence of donor‐type and acceptor‐type dopant‐level charge transfer and transfer within the interface of the ZnO/Co_3_O_4_ heterojunction.^[12]^ ZnO NPs showed a characteristic near‐band emission (NBE) at the wavelength of 390 nm, which is associated with direct Xenon light‐induced electron‐hole recombination.^[17]^ The NBE emission intensity was diminished for CDZO heterostructure. The intense defect‐related bands at 415 and 435 nm are due to the violet and blue emission, respectively. This violet‐blue emission is associated with the Zn vacancy (VZn) and host band electron transition.[^22^, ^23^] The CDZO heterostructure PL spectrum showed peak shifts towards higher wavelengths than single ZnO. This shift results from host‐sp and dopant‐d state‐localised electron‐strong exchange interactions.[^11^, ^16^] Besides, the intensity of the UV emission band decreased, and the broad visible emission band (467 nm) increased due to the increase in defects related to Co doping.[^17^, ^18^] The deconvoluted spectra of ZnO NPs and CDZO heterostructure clearly show intensity differences (Figure 2(e, f).

The morphological and elemental analysis by FESEM and EDS are illustrated in Figure 3(a, b). CDZO heterostructure (Figure 3a) shows a sponge‐like porous morphology due to the evolution of gases forming many pores.[^24^, ^25^] The EDS images (Figure 3(b, b1)) show a good Co distribution on the ZnO NP surfaces. Figure 3(b2–b4) shows Zn, Co, and oxygen (O) mapping images. Figure 3(b1) is the electron image from which the EDS layered image was taken.

Figure 3(b2–b4) shows Zn, Co, and oxygen (O) mapping images. The EDS elemental and compositional spectrum (Figure S1) of CDZO shows the presence of only Co, O, and Zn elements without any impurities. The TEM, HRTEM, and SAED ring images of CDZO are illustrated in Figure 3(c–e). The TEM image shows agglomerated particles with a 25 and 55 nm size range. The image also indicates porosity, which occurs as gas evolves during combustion. The d‐spacing value of 0.28 nm on the HRTEM image is associated with the ZnO NPs crystal (Figure 3d). No other d‐spacing value for Co_3_O_4_ was obtained. The inset FFT and IFFT images in Figure 3(d) represent the fast Fourier transform and the inverse fast Fourier transform, respectively. The concentric circles and bright spots on the SAED ring image indicate CDZO′s crystalline nature.

Photocatalytic dye degradation occurs after the pollutant is adsorbed on the surface of a catalyst. When exposed to light, electron‐hole pairs are generated and move on the surface, leading to a redox reaction. The crystallinity of the material and the amount of dopant are crucial factors for the photocatalytic degradation of dyes. Higher crystallinity enhances the degradation potential.^[26]^ Lu *et al*.^[27]^ investigated the impact of different amounts of Co dopant on photocatalytic dye degradation. It was found that the degradation activity increased with an increase in dopant amount up to a certain point, after which it decreased due to the accumulation of a new phase on the surface, which reduced the active sites and hindered charge migration. With this, the 15CDZO heterostructure, which had optimal crystallinity and the highest dopant amount, was chosen for further testing in photocatalysis. It is essential to increase the number of active surface sites (porosity) and to control surface charge for effective pollutant adsorption. Therefore, adjusting the pH of the solution to control and optimize the catalyst‘s surface charge is crucial.

**Figure 4:**
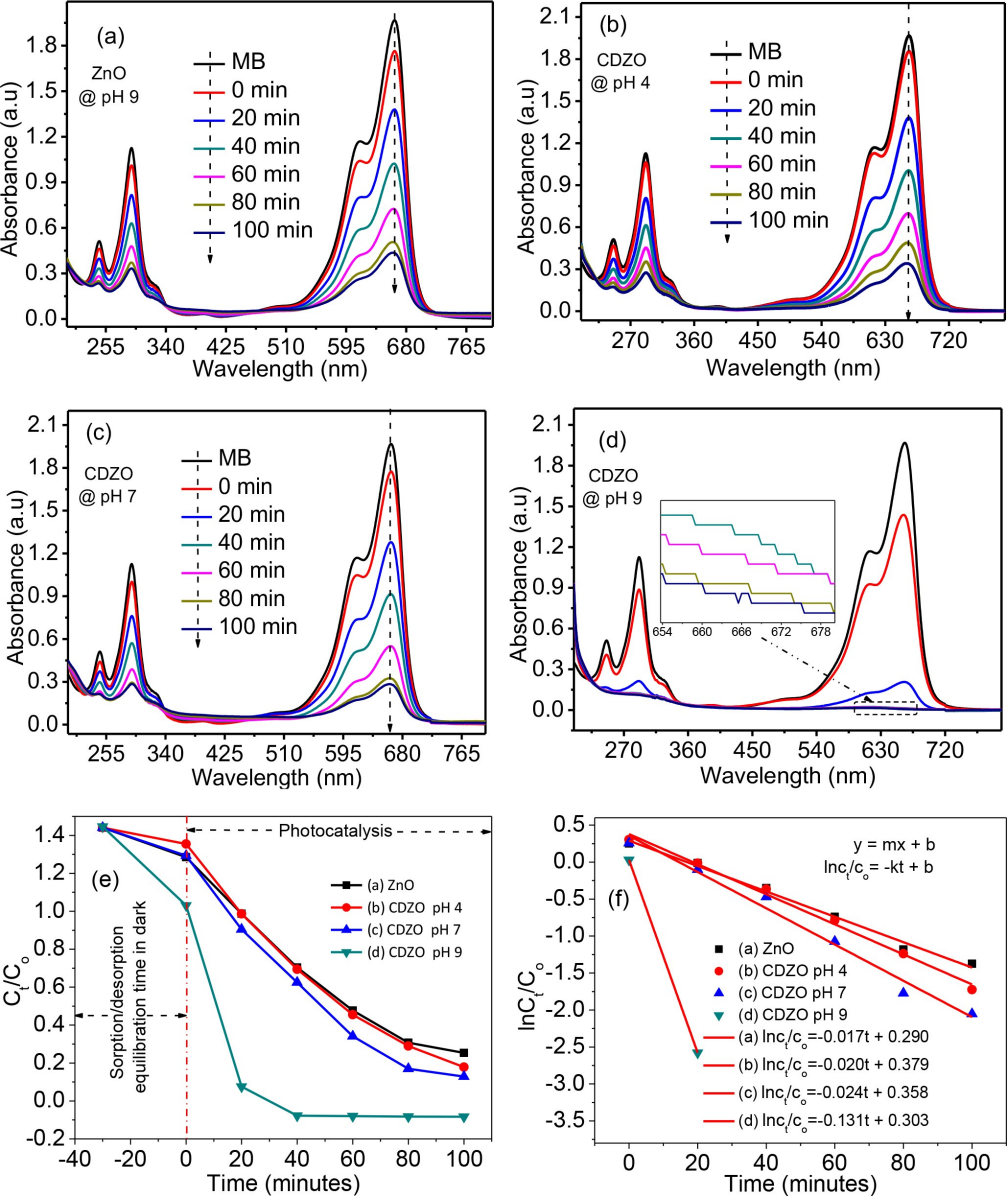
(a–d) absorbance versus wavelength plots, (e and f) C_t_/C_o_ and ln C_t_/C_o_ versus time photocatalytic analysis results for ZnO NPs and CDZO heterostructure.

**Figure 5:**
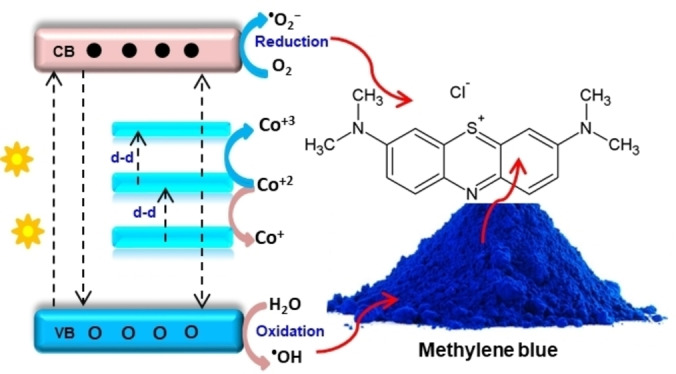
Different electron transitions are associated with the new cobalt 3d energy level, which helps boost methylene blue dye degradation.

The pH was optimized by testing different pH values of 4, 7, and 9 (Figure 4(b,c)). The photocatalytic potential was compared using rate constants (*k* values) (see Figure 4e, f). The determined *k* values for CDZO at pH 4, 7, and 9 are 0.020, 0.024, and 0.131 min^−1^, respectively. The catalyst surface becomes positively charged at a lower solution pH of 4 due to hydrogen ions. Conversely, at pH 9, the catalyst surface becomes negatively charged due to hydroxyl ions. Since the charge of methylene blue is positive, there is a repulsive electrostatic interaction at acidic pH and an attractive interaction at basic pH between the dye and the catalyst. This explains the significant differences in photocatalytic potential observed at pH 9 compared to pH 4 and 7. The greater attraction in the basic media is also supported by the superior sorption properties of CDZO heterostructure in the dark before irradiation (Figure 4(d, e)). The potential of CDZO heterostructure (0.131 min^−1^) is much higher than that of ZnO NPs (0.017 min^−1^) at the same pH 9 (Figure 4a). Furthermore, the photocatalytic degradation potential of the CDZO heterostructure surpasses that reported in the literature,[^28^, ^29^, ^30^] which has lower rate constant values than the present study (0.131 min^−1^). This improved photocatalytic potential for the CDZO heterostructure is attributed to enhanced light absorption from creating new Co 3d energy levels and the charge transfer properties between ZnO and Co_3_O_4_ (Figure 5).

The approximate valence band (VB) and conduction band (CB) edges of p‐type Co_3_O_4_ are +2.44 V and +0.37 V, respectively.[^31^, ^32^] The respective VB and CB edges of the n‐type ZnO semiconductor are 2.12 and −0.82.^[33]^ Once the ZnO/Co_3_O_4_ type II heterojunction forms, electrons can migrate from the more negative ZnO CB to the less negative Co_3_O_4_ CB. The holes migrate from the more positive Co_3_O_4_ VB to the less positive ZnO VB. In this mechanism, the electrons and holes collect on the low‐reducing and oxidizing energy. Besides, their movement is also disrupted due to the host‐guest electron‐hole repulsion. Thus, either the Z‐ or S‐scheme is the most probable mechanism for the superior dye degradation potential of Co‐ZnO/Co_3_O_4_. In the Z‐ or S‐scheme mechanism, CB electrons of Co_3_O_4_ recombine with ZnO VB holes. The ZnO electrons and Co_3_O_4_ VB holes with high thermodynamic energy react with oxygen and water, generate reactive species, and degrade MB dye.[^34^, ^35^]

## Conclusions

The combustion method created a Co‐doped ZnO nanoparticle heterostructure with good porosity. This doped heterostructure has improved optical properties and increased absorption of visible light. The three bands in the visible region of the DRS‐UV‐vis spectrum are associated with specific d–d transitions. The indirect bandgap for ZnO NPs and CDZO heterostructure was found to be 3.1 and 1.8 eV, respectively. The porosity and even distribution of Co on the surface, which are essential for catalytic applications, have been confirmed by FESEM and EDS elemental mapping analyses. The TEM image also confirmed the presence of porosity within the NPs formed due to gas evolution. The size of the NPs is 25–55 nm. The heterostructure is crystalline, as confirmed by the SAED ring and bright spots. The PL spectra showed reduced emission intensity and a higher wavelength shift for the CDZO heterostructure than ZnO NPs. The reduction in intensity is associated with the hindrance of electron‐hole recombination in the CDZO heterostructure, while the transfer is due to the sp‐d exchange interaction. The enhanced photocatalytic property of CDZO heterostructure (0.131 min^−1^) over ZnO NPs (0.017 min^−1^) is due to the extended visible light absorption and charge transfer properties through the ZnO/Co_3_O_4_ interface.

## Experimental Section

### Synthesis of Catalyts

A single‐source dopant diffusion procedure (nitrate salt precursor)^[19]^ and the hard‐soft acid‐base principle^[20]^ were employed to select soft acid (cobalt), which can easily diffuse in the borderline ZnO NP host. The soft acid dopant can easily diffuse and substitute for the borderline Zn ions. The salts were mixed in pre‐dissolved polyvinyl alcohol (PVA) aqueous solutions.^[36]^ Specifically, 1.00 g of PVA was first dissolved in distilled water at 80 °C^[37]^. The precursor‐PVA solution complex was then dehydrated to form a gel at 105 °C and combusted at 155–265 °C to produce a porous product. The product was subsequently calcined in a furnace at 450 °C for 1 hour. Different percentages of Co dopant (0.5 %, 5 %, 15 %, and 30 %) were synthesized using the abovementioned procedure.

### Photocatalytic Dye Degradation

The synthesized porous heterostructures were characterized and utilized for photocatalysis applications. Under testing conditions using a Xenon lamp with a wide wavelength range of 100–800 nm, the photocatalytic properties of ZnO NPs and a selected CDZO heterostructure were evaluated. A concentration of 10 mg/L of methylene blue dye (MB) was used as a synthetic pollutant, and a 20 mg catalyst dosage was applied. The reactor temperature is controlled using water circulation and electric fans. The pH of the solutions, which is crucial in controlling surface charge, was optimized by testing different dye‐catalyst solutions with pH of 4, 7, and 9. The photocatalytic potential was measured at a 20‐minute interval using a UV‐vis spectrometer.

## Conflict of Interests

The authors declare no conflict of interest.

1

## Supporting information

As a service to our authors and readers, this journal provides supporting information supplied by the authors. Such materials are peer reviewed and may be re‐organized for online delivery, but are not copy‐edited or typeset. Technical support issues arising from supporting information (other than missing files) should be addressed to the authors.

Supporting Information

## Data Availability

The data that support the findings of this study are available from the corresponding author upon reasonable request.
