# Supplementary material for: Cobalt‐Doped ZnO Nanocomposits for Efficient Dye Degradation: Charge Transfer
Source: ChemistryOpen. 2024 Sep 9;13(12):e202400203. doi: 10.1002/open.202400203 (PMC11625955; doi:10.1002/open.202400203)
Supplement: Supplementary file 1 — Supporting Information [file OPEN-13-e202400203-s001.pdf]

ChemistryOpen

Supporting Information

Cobalt-Doped ZnO Nanocomposits for Efficient Dye Degradation: Charge Transfer

Buzuayehu Abebe,* Bontu Kefale, Guta Amenu, Leta Guta, C. R. Ravikumar, Taymour A. Hamdalla, S. Giridhar Reddy, Dereje Tsegaye,* and H. C. Ananda Murthy

Supporting Information

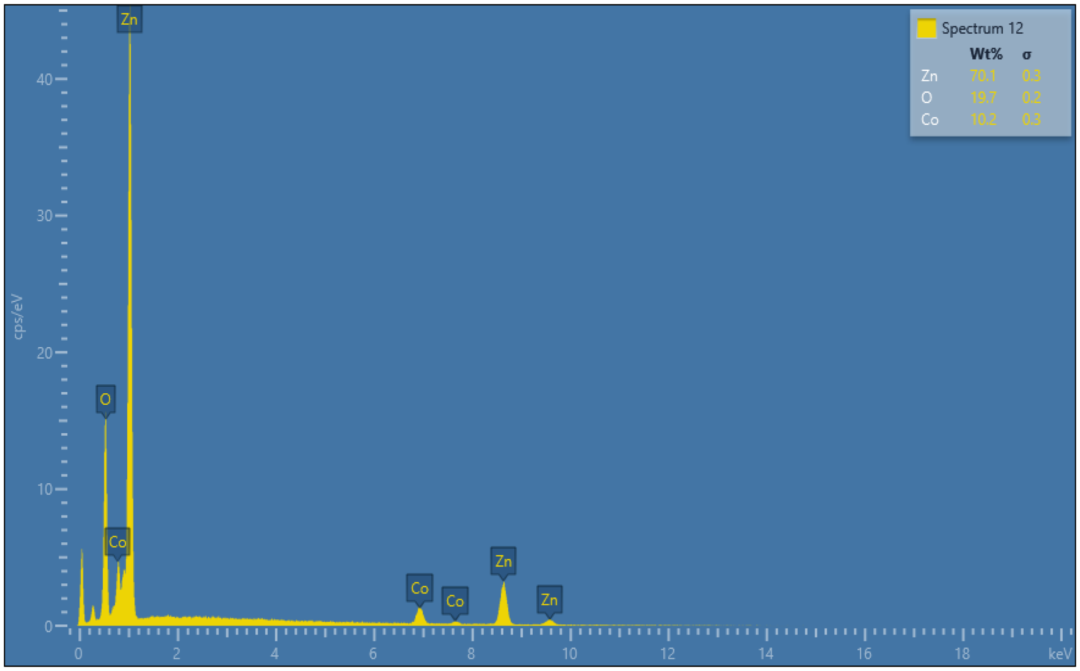

Figure S1. the EDX elemental and compositional analysis spectra of CDZO nanomaterial.
